# Control of Graves’ hyperthyroidism with very long-term methimazole treatment: a clinical trial

**DOI:** 10.1186/s12902-020-00670-w

**Published:** 2021-01-14

**Authors:** Fereidoun Azizi, Hengameh Abdi, Atieh Amouzegar

**Affiliations:** Endocrine Research Center, Research Institute for Endocrine Sciences, Shahid Beheshti University of Medical Sciences, P.O.Box: 19395-4763, 1985717413, No 24, Aerabi St, Daneshjoo Blv, Velenjak, Tehran, Iran

## Abstract

**Background:**

Long-term antithyroid drug therapy has become one of the options for treatment of Graves’ hyperthyroidism. The aim of this study was to compare thyroid status in those who discontinued methimazole (MMI) treatment after 12.8 years with those who continued MMI as long as 24 years.

**Methods:**

Fifty nine patients with Graves’ disease on long-term MMI for 14.2 ± 2.9 years were recruited; 32 patients (54%) decided to discontinue MMI and 27 (46%) preferred additional years of MMI treatment. All patients were followed for a mean of 6 additional years.

**Results:**

Of 27 patients who continued MMI up to 24 years, suppressed serum thyrotropin (TSH) was not observed in any patient after the seventh year of treatment. Serum free thyroxine, triiodothyronine, TSH and TSH receptor antibody concentrations remained normal up to the length of the study. Mean daily dose of MMI to maintain TSH in the reference range decreased gradually and reached to 2.8 ± 1.7 mg by 24 years of MMI treatment. No adverse reaction related to MMI occured during additional years of therapy. In 32 patients who discontinued MMI, hyperthyroidism relapsed in 6 patients (19%), one left follow-up and 25 (78%) remained euthyroid during the study.

**Conclusions:**

Long-term low dose MMI treatment may be a lifelong effective and safe therapeutic modality in patients with Graves’ hyperthyroidism for prevention of relapse, if studies from other centers confirm findings of this research.

**Trial registration:**

IRCT201009224794N1, 2010-10-25. Retrospectively registered.

https://www.irct.ir/trial/5143.

## Introduction

Antithyroid thionamide drugs (ATDs) have become the treatment of choice for Graves’ disease (GD) in the United States, and majority of patients in other countries worldwide [1–3]. The major drawback of ATD therapy is its 20–70% recurrence rate of hyperthyroidism following discontinuation of the traditional 12–18 month treatment [4]. Serum thyrotropin (TSH) receptor antibody (TRAb) concentration, one major predictor of GD relapse, may fluctuate or remain positive in many patients with GD following ATD withdrawal and median time to remission may last as long as 6.8 years [5]. Hence, a few studies from different regions of the world have adopted the long-term continuous ATD therapy [6–10]; based on findings of a meta-analysis, remission rate increases by 16% for each additional year of ATD therapy after 24 months of ATD treatment [11]. These findings indicate that failure to attain normal TRAb concentrations after conventional 12–18 months of ATD treatment does not rule out the possibility of remission over a long-term ATD therapy [12]; therefore, long-term treatment of Graves’ hyperthyroidism has become an option in international societies recommendations [4, 13]. A recent randomized clinical trial reported that 5-year continuous methimazole (MMI) therapy is accompanied with 84% remission up to 4 years after drug withdrawal [14]. However, the optimal duration of ATD therapy is still debatable. Likewise, details of clinical and biochemical changes in those with ATD treatment > 10–15 years are scarce. In the present study, we aimed to compare thyroid status in patients who discontinued MMI treatment after 12.8 years with those who continued MMI therapy for as long as 24 years.

## Methods

The study protocol was approved by the ethics committee of the Research Institute for Endocrine Sciences and all patients gave written informed consent.

This study is an extension of long-term methimazole (LT-MMI) treatment of patients with GD [7]. From March 1987, all patients with recurrent GD who had ongoing long-term methimazole (LT-MMI) therapy, as part of a clinical trial entitled “*Towards Outstanding Hyperthyroid care Induced by anthithyroid Drugs*” (TOHID study), registered in the Iranian Registry of Clinical Trials (www. IRCT.IR/Trial/5143) were recruited. The aim of previous study was to compare the effectiveness of long-term continuous MMI therapy with radioiodine (RAI) treatment in patients with GD; 239 patients with recurrence of hyperthyroidism after a conventional 12–18 months of ATD treatment were divided into MMI and RAI groups. The diagnosis of Graves’ hyperthyroidism was based on clinical findings of hyperthyroidism with or without Graves’ orbitopathy, serum TSH < 0.4 mU/L, free thyroxine (fT4) > 23 pmol/L and/or serum triiodothyronine (T3) > 200 ng/dL, elevated TRAb levels > 1.75 IU/L and diffuse goiter without nodularity on technetium scintigraphy. Patients were prescribed 20–30 mg MMI for the first month and titration method was used to maintain serum fT4 between 10 and 23 pmol/L and serum TSH between 0.4–5.0 mU/L. Patients were visited monthly for the first 3 months of therapy and every six months thereafter by the principle investigator (F.A.). During each visit, complete history and a review of symptoms were documented and physical examination, in particular related to thyroid size and its function was performed. All possible adverse effects due to ATD therapy were ascertained. Cell blood count, serum levels of fT4, T3, TSH, alanine aminotransferase and aspartate aminotransferase were measured at baseline. At each visit, the dose of methimazole was adjusted to maintain serum fT4 and T3 concentration in the middle range of normal values and TSH in the reference range.

## Procedures

**Fig. 1:**
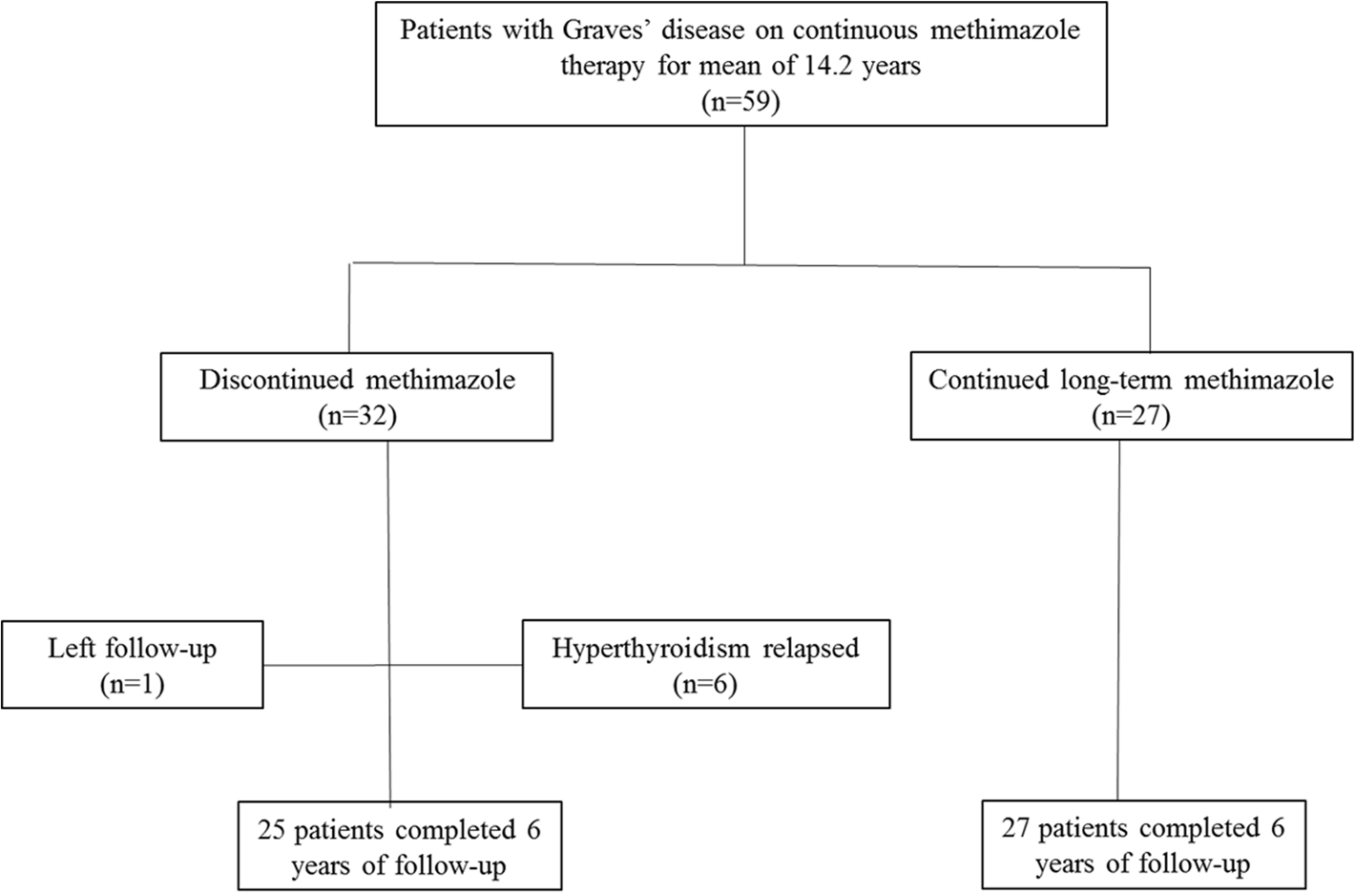
Enrollment and follow-up of study patients

For this study, all 59 patients who had been on LT-MMI treatment for 14.2 ± 2.9 years were recruited; the principle investigator (F.A.) explained about advantages and disadvantages of MMI withdrawal versus MMI continuation for additional years, doing his best not to influence on the patient choice. They were given the choice of either discontinuing MMI treatment or continue LT-MMI therapy; 32 patients (54%) decided to discontinue MMI and 27 (46%) preferred continuous LT-MMI treatment (Fig. 1). All patients were followed every six months, as described above.

## Laboratory measurements

Before 2005, serum fT4 and T3 were measured by radioimmunoassay kits from DiaMetra, Milan, Italy and serum TSH by immunoradiometric assay using kits from Izotop (Budapest, Hungary). Serum TRAb concentration was measured by enzme-linked immunoabsorbent assay (Bio Vendor Laboratory Medicine Inc., Czech Republic). Subsequently, all analyses were determined by electrochemiluminescence immunoassay (Roche Diagnostic GmbH, Mannheim, Germany). Interassay and intra-assay coefficients of variation of all tests were < 6.1% and < 9.1%, respectively.

## Definitions

Thyroid function status was defined as follows: Euthyroidism, TSH 0.4–5.0 mU/L; hypothyroidism, TSH > 5.0 mU/L and fT4 < 9 pmol/L; subclinical hypothyroidism, TSH > 5.0 mU/L and fT4 9–23 pmol/L; hyperthyroidism, TSH < 0.4 mU/L and fT4 > 23 pmol/L and/or T3 > 200 ng/dL and subclinical hyperthyroidism, TSH < 0.4 mU/L and fT4 9–23 pmol/L and T3 80–200 ng/dL.

## Outcomes

The primary outcome of the study was sustained euthyroidism during the additional six years of follow-up. Key secondary outcomes were the occurrence of both clinical and subclinical hyper- and hypothyroidism during the length of study. Assessment of safety of MMI therapy was performed by observation of adverse events during the treatment.

## Statistical analysis

Data are reported as mean ± SD for continuous and number (percentages) for categorical variables. Significant differences were assessed by Student’s t, Mann-Whitney, Chi-square and Fisher exact tests. Change of continuous variables during very long-term MMI treatment was analyzed using generalized estimating equation. Time to relapse of hyperthyroidism after MMI withdrawal was compared using Kaplan-Meier curves and log-rank test was used to compare survival curves. Statistical analysis was performed by SPSS 20 (SPSS Inc., Chicago, IL) and *p* < 0.05 was considered significant.

## Results

Baseline characteristics of two study groups are shown in Table 1. There were no significant differences in age, sex distribution, smoking status, goiter size and serum concentrations of fT4, T3, TSH and TRAb and daily MMI dosage at the time of this study entry between those who continued versus stopped MMI treatment. Nonetheless, patients who continued MMI therapy had received MMI for longer duration as compared to those who discontinued MMI (15.6 ± 1.9 vs. 12.8 ± 4.0 years, respectively).

**Table 1:** Characteristics of two study groups at the start of the current study

Variables	Continued MMI (***n*** = 27)	Discontinued MMI (***n*** = 32)
Age, year	53.1 ± 19.8	49.7 ± 12.4
Female, *n* (%)	19 (70)	22 (69)
High school diploma, *n* (%)	11 (41)	12 (38)
Goiter grade^a^, *n* (%)		
0 or 1	9 (33)	10 (31)
2	18 (67)	22 (69)
Current smoking, *n* (%)	4 (15)	5 (16)
Ophthalmopathy, *n* (%)	4 (15)	5 (16)
fT4, pmol/L	16.1 ± 2.4	15.8 ± 2.6
T3, ng/dL	125 ± 21	132 ± 26
TSH, mIU/L	3.0 ± 0.6	2.9 ± 1.2
TRAb, IU/L^b^	1.3 ± 0.6	1.1 ± 0.7
MMI dose, mg/day	3.4 ± 1.1	3.8 ± 1.2
Duration of MMI therapy, years^‡^	15.6 ± 1.9	12.8 ± 4.0
Comorbidities^c^, *n* (%)	7 (26)	9 (28)

Abbreviations: *MMI* methimazole; *fT4* free thyroxine; *T3* triiodothyronine; *TSH* thyrotropin, *TRAb* TSH receptor antibody

^a^Goiter size: Grade 0, thyroid is not palpable or visible; grade I, thyroid is easily palpable but not visible; grade 2, thyroid easily visible with the head in the normal position

^b^Serum TRAb concentration was below 2.3 IU/L in all patients

^c^Comorbidities included documented diabetes, hypertension, or dyslipidemia

^‡^*P*-value < 0.001

### Continued MMI group

**Fig. 2:**
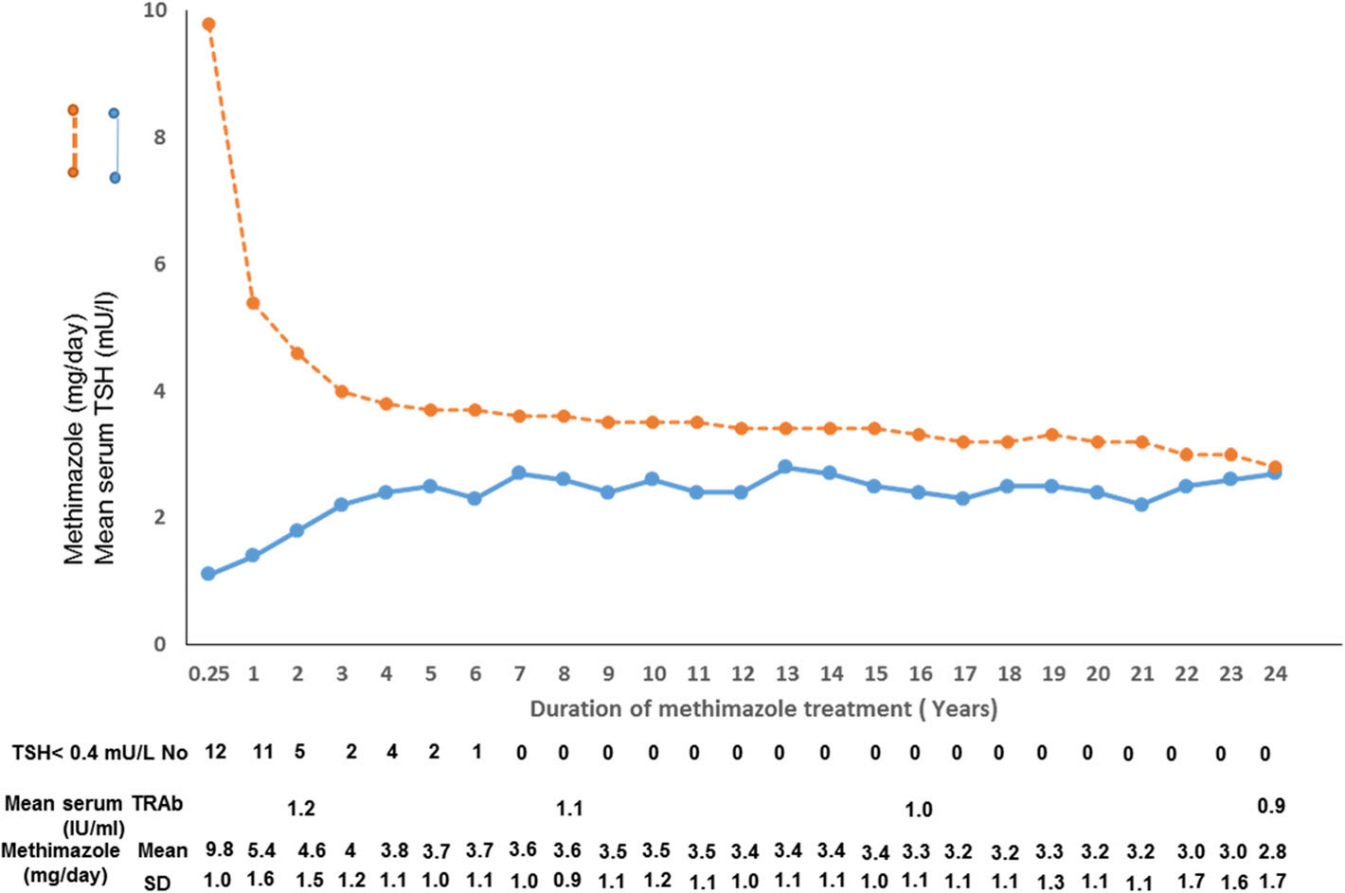
Serum concentrations of TSH and TSH receptor antibody (TRAb) and mean daily doses of methimazole during 24 years of continuous methimazole treatment. All 27 patients continued therapy for at least 15 years, 16 patients until 20 years and 11 patients until 24 years. Daily doses of methimazole to maintain euthyroidism decreased to mean of 3.4 ± 1.0 and 2.8 ± 1.7 mg daily, by 15 and 24 years of therapy; suppressed serum TSH was not seen in any patient after 7 years of treatment. Serum TRAb was normal in all patients during methimazole treatment

At the start of the current study, 27 patients including 19 females and 8 males of this group were 53.1 ± 19.8 years old and have already been on 15.6 ± 1.9 years of LT-MMI therapy. Figure 2 shows serum concentrations of TSH and MMI dosages used to maintain euthyroidism during 24 years of continuous LT-MMI treatment. After 3 months of MMI treatment, serum TSH increased to normal range in 12 (44%) of patients. By 4 years after LT-MMI, only 4 (15%) had at least one suppressed TSH during the follow-up. Seven years after the start of LT-MMI, no suppressed TSH was observed and TSH remained stable and in normal range in all patients up to 24 years following MMI treatment (*p* = 0.32). Serum fT4 decreased from a mean of 39.1 ± 9.2 pmol/L at the baseline to 16.1 ± 25, 16.3 ± 2.4 and 16.2 ± 2.2 pmol/L at 15, 20 and 24 years after LT-MMI treatment; the decreasing trend of fT4 concentration from the first to the 24th year of treatment was significant (*p* < 0.001). Mean serum T3 concentration decreased from 401 ± 126 ng/dL to 125 ± 18, 123 ± 17 and 124 ± 17 ng/dL at 15, 20 and 24 years of treatment; the decreasing trend of T3 from the first to the 24th year of treatment was also significant (*p* < 0.001) (Table 2). Mean serum concentration of TRAb was 15 ± 8 IU/ml at baseline and decreased to 1.2, 1.1, 1.0 and 0.9 IU/ml after 2, 8, 16 and 24 year of LT-MMI therapy (*p* < 0.001, Fig. 2). After 6 years of continuous MMI therapy, none of the patients had TRAb levels above 1.7 IU/ml.

**Table 2:** Serum concentrations of thyroid hormones, TSH and TRAb in Graves’ patients during 24 years of continuous long-term MMI treatment

	Baseline	Years of very long-term MMI treatment					
		1	5	10	15	20	24
Number	27	27	27	27	27	16	11
Serum fT4 (pmol/L)^*^	39.1 ± 9.2	16.9 ± 2.3	16.3 ± 2.3	16.2 ± 2.4	16.1 ± 2.5	16.3 ± 2.4	16.2 ± 2.2
Serum T3 (ng/dL)^*^	401 ± 126	135 ± 18	127 ± 18	129 ± 21	125 ± 18	123 ± 17	124 ± 17
Serum TSH (mU/L)^†^							
Mean ± SD	All < 0.1	1.5 ± 1.6	2.5 ± 1.6	2.6 ± 1.6	2.5 ± 1.0	2.4 ± 1.2	2.7 ± 0.7
Median (IQR)	All < 0.1	0.6 (0.2–2.4)	2.4 (1.0–3.1)	2.5 (1.5–3.3)	2.5 (1.9–3.1)	2.5 (1.8–2.9)	2.8 (2.6–3.1)
Serum TRAb (IU/mL)^†^							
Mean ± SD	14.0 ± 7.0	1.2 ± 0.7	1.2 ± 0.6	1.1 ± 0.6	1.0 ± 0.6	1.0 ± 0.5	0.9 ± 0.4
Median (IQR)	–	1.2 (0.6–1.4)	–	1.1 (0.5–1.4)	1.0 (0.4–1.3)	–	0.9 (0.4–1.2)

^*^Values are mean ± standard deviation

^†^Normally distributed values. Serum concentrations of TSH and TRAb were in normal range after 6 years of treatment

*fT4* free thyroxine, *T3* triiodothyronine, *TSH* thyrotropin, *TRAb* TSH receptor antibody, *MMI* methimazole

Mean dose of MMI was 9.8 ± 1.0 mg daily after three months of therapy and decreased to 5.4 ± 1.6 mg per day by the end of the first year of MMI treatment. Daily doses of MMI to maintain euthyroidism were gradually lowered to ≤5 mg by 10 years after the start of study in all patients. Mean daily dose of MMI decreased to 2.8 ± 1.7 mg daily after 24 years of LT-MMI treatment (Fig. 2); 5 of 11 patients required ≤2.5 mg daily MMI to remain euthyroid. None of the subjects showed recurrence on therapy. Six patients developed minor skin reactions in the first few months of therapy; adverse reactions such as agranulocytosis and liver dysfunction were not observed. Thereafter, no adverse reactions related to MMI occurred during 24 years of follow-up.

### Discontinued MMI group

**Fig. 3:**
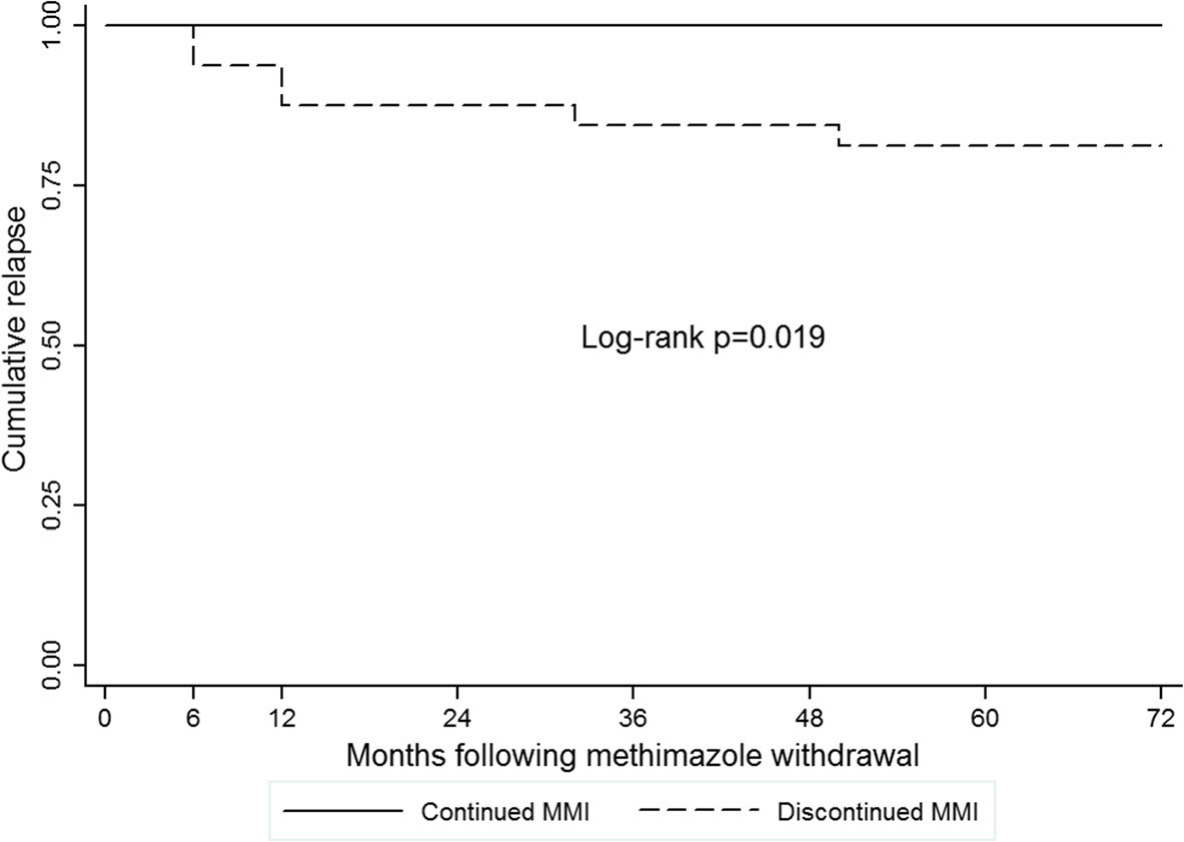
Kaplan-Meier curve for relapse of hyperthyroidism in patients with Graves’ disease after long-term methimazole (MMI) treatment. No recurrence was observed in patients who continued MMI; hyperthyroidism relapsed in 19% of those who discontinued MMI therapy

Twenty-two females and 10 males, aged 49.7 ± 12.4 years who were on LT-MMI for 12.8 ± 4.0 years, decided to discontinue MMI treatment. Trends of changes in serum concentrations of fT4, T3, TSH, TRAb and MMI dosage during 12.8 years of MMI treatment was not different from patients who decided to continue MMI treatment. Of these 32 patients, one left follow-up; overt hyperthyroidism relapsed in six patients during 6 years of follow-up. Two, two, one and one patients recurred at 6, 12, 32 and 50 months after discontinuation of MMI treatment. Other 25 (78%) patients in this group had normal serum concentrations of fT4, T3 and TSH and serum TRAb < 1.7 IU/ml during 6 additional years of follow-up. Figure 3 shows Kaplan-Meier curve for relapse of hyperthyroidism in patients who continued and those who discontinued MMI after 6 years of follow-up. There was no relapse in the first group and 19% relapse in the second group (log rank *p* = 0.019).

## Discussion

The present study demonstrates continuous decline in serum fT4 and T3 concentrations and rise in serum TSH during 24 years of MMI treatment accompanied by continuous normalization of serum TRAb and gradual decrease in daily doses of MMI to maintain euthyroid state. We have not found any episode of exacerbation of hyperthyroidism during 24 years of follow-up in patients treated with continuous LT-MMI treatment. In addition, no adverse events occurred after the first year up to 24 years of continuous MMI treatment; findings in agreement with a recent systematic review on safety of long-term ATD treatment [15].

Several studies have reported that in patients who experience relapse of hyperthyroidism after 12–18 months of ATD therapy, remission may be attained by additional 5 to 10 years of treatment [8, 11, 14] Therefore, some patients may prefer continuous lifelong MMI therapy for management of Graves’ hyperthyroidism. In the present study, majority of patients who discontinued treatment after mean 12.8 years of continuous MMI stayed euthyroid during 6 years of follow-up; only 19% experienced relapse of hyperthyroidism. Results are similar to other studies reporting increased remission rates in Graves’ hyperthyroidism after long-term MMI therapy, compared to the conventional 12–18 month ATD therapy [11]. A proposed justification for the observed results of LT-MMI therapy is related to immunomodulatory properties of MMI, an effect which is not yet fully understood [16].

The 2016 American Thyroid Association guidelines recommend RAI or thyroidectomy as the therapeutic choices in the setting of hyperthyroidism after the conventional 12–18 months of ATD therapy; continuous low dose MMI has been suggested in patients with persistently high TRAb concentrations [4]. Safety of LT-ATD treatment has been shown in clinical studies [15]. Likewise, based on the findings of a recently published trial, LT-ATD treatment of ≥5 years induces cure of disease in 86% of patients with GD after ATD discontinuation [14]. In a large cohort of GD patients treated by ATD from Japan, Okamura, et al. showed that serum TRAb level may become normal following the conventional ATD treatment but it will fluctuate and remission of GD occurs after the median 6.2 years of ATD therapy [17]. These findings point to the importance of LT-ATD treatment to ensure higher probability of remission.

The beneficial effect of LT-ATD therapy has been documented in patients with Graves’ orbitopathy [9] and those with relapse of hyperthyroidism following conventional 12–18 months ATD treatment [6, 8]. It has also been recommended in patients with high serum TRAb concentration at the end of 18 months course of ATD therapy [4]. Our experience in the present study shows that even in those with continuous positive TRAb, LT-MMI treatment causes gradual decrease of serum TRAb concentrations and remission of hyperthyroidism. Previous studies have also shown more stable euthyroidism and comparable or better cost-benefit when compared to RAI treatment [6]. Considering recent findings that effective control of hyperthyroidism improves cardiovascular outcomes [18], a stable euthyroid state during continuous MMI treatment highlights a potential advantage of this mode of therapy.

Strengths of this study are the longest follow-up of patients with continuous MMI therapy and demonstration of the point of normalization of TSH in all treated patients. In addition, findings emphasize the fact that many patients require < 2.5 mg MMI daily to remain euthyroid and there is paucity of adverse events on low dose LT-MMI treatment. Following limitations are noteworthy; first, the study arm was selected by each patient choice; therefore, study enrollment was not randomized and possibilities of biases may exist which can affect the results. Second, with regards to the proposed relationships between iodine intake and efficacy of ATDs, although still unresolved and contradictory [19], our findings in an iodine-sufficient population may not be generalizable to other regions. Third, those who continued very LT-MMI treatment were already 2.8 years more on MMI than patients who discontinued medication.

In conclusion, long-term low dose MMI treatment may be prescribed effectively, even throughout the patients’ life for those with Graves’ hyperthyroidism who do not desire ablation treatment. Low cost, safe and effective drugs are prescribed as lifelong therapy for some specific diseases, such as epilepsy, inflammatory bowel disease and hypothyroidism and MMI may be added to the list of lifelong drugs for control of Graves’ hyperthyroidism. During long-term MMI therapy in women of reproductive age, special attention should be made to discontinue MMI or change to propylthiouracil in those planning for pregnancy [20]. It is noteworthy that final decision to select mode of treatment in GD is according to physicians and patients decisions and some may prefer definitive therapy of hyperthyroidism by ablation over long-term treatment. In addition, the findings of the present article are from a single-center study by investigators with expertise in low dose MMI therapy. Further very long-term ATD treatment studies from other clinical centers with more sophisticated designs in order to minimize biases will shed more light on the efficacy of this mode of therapy in GD and perhaps other causes of hyperthyroidism.

## Data Availability

The datasets generated and/or analysed during the current study are not publicly available due to repository at a private clinic but are available from the corresponding author on reasonable request.
